# A Mobile Health Contraception Decision Support Intervention for Latina Adolescents: Implementation Evaluation for Use in School-Based Health Centers

**DOI:** 10.2196/11163

**Published:** 2019-03-14

**Authors:** Kathleen P Tebb, Sang Leng Trieu, Rosario Rico, Robert Renteria, Felicia Rodriguez, Maryjane Puffer

**Affiliations:** 1 Department of Pediatrics University of California, San Francisco San Francisco, CA United States; 2 The Los Angeles Trust for Children's Health Los Angeles, California, CA United States

**Keywords:** mobile health, adolescent health, pregnancy in adolescence

## Abstract

**Background:**

Health care providers are a trusted and accurate source of sexual health information for most adolescents, and clinical guidelines recommend that all youth receive comprehensive, confidential sexual health information and services. However, these guidelines are followed inconsistently. Providers often lack the time, comfort, and skills to provide patient-centered comprehensive contraceptive counseling and services. There are significant disparities in the provision of sexual health services for Latino adolescents, which contribute to disproportionately higher rates of teenage pregnancy. To address this, we developed *Health-E You* or *Salud iTu* in Spanish, an evidence-informed mobile health (mHealth) app, to provide interactive, individually tailored sexual health information and contraception decision support for English and Spanish speakers. It is designed to be used in conjunction with a clinical encounter to increase access to patient-centered contraceptive information and services for adolescents at risk of pregnancy. Based on user input, the app provides tailored contraceptive recommendations and asks the youth to indicate what methods they are most interested in. This information is shared with the provider before the in-person visit. The app is designed to prepare youth for the visit and acts as a clinician extender to support the delivery of health education and enhance the quality of patient-centered sexual health care. Despite the promise of this app, there is limited research on the integration of such interventions into clinical practice.

**Objective:**

This study described efforts used to support the successful adoption and implementation of the *Health-E You* app in clinical settings and described facilitators and barriers encountered to inform future efforts aimed at integrating mHealth interventions into clinical settings.

**Methods:**

This study was part of a larger, cluster randomized control trial to evaluate the effectiveness of *Health-E You* on its ability to reduce health disparities in contraceptive knowledge, access to contraceptive services, and unintended pregnancies among sexually active Latina adolescents at 18 school-based health centers (SBHCs) across Los Angeles County, California. App development and implementation were informed by the theory of diffusion of innovation, the Patient-Centered Outcomes Research Institute’s principles of engagement, and iterative pilot testing with adolescents and clinicians. Implementation facilitators and barriers were identified through monthly conference calls, site visits, and quarterly in-person collaborative meetings.

**Results:**

Implementation approaches enhanced the development, adoption, and integration of *Health-E You* into SBHCs. Implementation challenges were also identified to improve the integration of mHealth interventions into clinical settings.

**Conclusions:**

This study provides important insights that can inform and improve the implementation efforts for future mHealth interventions. In particular, an implementation approach founded in a strong theoretical framework and active engagement with patient and community partners can enhance the development, adoption, and integration of mHealth technologies into clinical practice.

**Trial Registration:**

ClinicalTrials.gov NCT02847858; https://clinicaltrials.gov/ct2/show/NCT02847858 (Archived by WebCite at http://www.webcitation.org/761yVIRTp).

## Introduction

### Background

Despite the widespread use of mobile technologies (mobile phones, tablets, and computers) among adolescents and the rapid proliferation of mobile health (mHealth) apps, few studies have examined how to implement and integrate such technologies into clinical settings.

Mobile technologies are an especially attractive medium for adolescents and can be a powerful tool to promote health. Specifically, adolescents use computers and access the internet more than any other age group [1], and the use of computers to deliver behavioral health interventions is rapidly expanding [2-5]. Furthermore, computer-based sexual health risk assessments have been found to be acceptable to adolescents and to improve the disclosure of sexual health risk behaviors [3-7] and address psychological aspects of behavior in ways that teens perceive to be less judgmental than advice from a health educator or clinician [7].

Mobile technologies can also serve as a clinician extender and overcome barriers to promoting sexual and reproductive health services for adolescents. Although health care providers are a trusted and accurate source of sexual health information for adolescents [8] and clinical guidelines recommend that all adolescents receive comprehensive, confidential sexual health information and services [9,10], these guidelines are followed inconsistently [11,12]. Providers often lack the time, comfort, and skills to provide patient-centered comprehensive contraceptive counseling and services [13-15]. Many youth lack access to comprehensive, confidential contraceptive information and services, and racial disparities in teenage pregnancy rates persist despite overall declines across the United States [16,17]. A few Web-based contraceptive decision support tools have been developed to improve adolescents’ and young adults’ knowledge and use of contraceptives [18-20]; however, these are primarily Web-based programs that require a user to take the initiative to seek out these resources and are often aimed at young adults rather than adolescents [21]. In addition, most Latino and heterosexual adolescents do not actively seek out sexual health information online [22,23]. Furthermore, although youth find the privacy, anonymity, and ease of access to such information appealing, there is a vast amount of inaccurate sexual health information on the internet [24].

### The Health-E You/Salud iTu App

To address this health care gap, we have developed an evidence-informed mHealth app, *Health-E You* or *Salud iTu* in Spanish, specifically for adolescent girls. This app is interactive, individually tailored, and provides patient-centered, contraceptive information and decision support in both English and Spanish. This is an internet-enabled app that can be used on a range of mobile devices (iPads, mobile phones, or computers). It is designed to be used in conjunction with a clinical encounter to support the contraceptive decision-making process; increase access to patient-centered, evidence-based contraceptive information and services; and ultimately reduce disparities in contraceptive knowledge, access to contraceptive information and services, and unintended pregnancies among Latina adolescents. The app was developed with significant input from Latina adolescents and health care providers. It is designed to individualize the educational experience by responding to a patient’s unique needs, attitudes, experiences, and risk profiles. The app provides tailored health information based on the user’s inputs and supports them in selecting a contraceptive method that is a good fit for them. When used in conjunction with a clinical visit, the app aims to (1) prepare adolescent patients for the visit and encourage them to ask about and, or, advocate for contraceptive services that are of interest to them and (2) act as a clinician extender to support the delivery of health education and enhance the quality of patient-centered care.

### Objectives

To date, there is limited evidence on how to best utilize mHealth technologies, such as this app, to support effective interactions between adolescent patients and health care providers in a clinical setting [25]. To our knowledge, there are only 2 studies that designed and evaluated a contraceptive decision support tool for use in a clinical setting [26,27]. Although both these studies improved contraceptive knowledge and use, they were focused on outcomes and provided little detail on *how* the app was implemented. In addition, 1 study was relatively old (published in 1999) and was limited to oral contraceptives only [26]. With the rise of mHealth interventions aimed at enhancing the delivery of health education and services, there is a tremendous need to better understand processes to ensure effective implementation and integration into the clinical context [28].The purpose of this paper is to describe efforts to improve the successful adoption and implementation of the *Health-E You* app in clinical settings and to identify implementation facilitators and barriers that may be relevant to other types of computer apps being integrated across different clinical settings.

## Methods

### Study Context

This study is part of a larger, cluster randomized control trial (CRCT) conducted at 18 school-based health centers (SBHCs) across Los Angeles County, California, to evaluate the effectiveness of *Health-E You* on its ability to reduce health disparities in unintended pregnancies among sexually active Latina adolescents [29]. All clinics are SBHCs that are operated and managed independently, cover a very large area of Los Angeles County, serve a large proportion of Latina students, and are centered in areas with high rates of sexual health morbidities according to the Los Angeles County Department of Public Health. The Institutional Review Board (IRB) for Protection of Human Subjects of the University of California, San Francisco, approved this study (IRB approval number: 10-02730). As this study is integrated into the delivery of sensitive, sexual health services provided in clinic, by the state law, these services are confidential and parental consent is not required. Parental consent to participate in the study was waived by the IRB to protect adolescent confidentiality and to comply with the state law.

### App Development and Pilot Testing

A youth-centered design approach [30] was used to develop and test the app. In this approach, youth are engaged in all aspects of design, prototyping and pilot testing, and revision to achieve the final design of *Health-E You*. Several focus groups of youth from the SBHCs were conducted to gather input on the original design of the app. Youth provided feedback on design attributes that they felt would resonate with their peers, including content, images, font, and design layouts. The app was pilot tested in 3 SBHCs, which showed that Latina adolescents found it acceptable and it improved their knowledge and intentions to use effective contraception, and health care providers and staff agreed that it was feasible to implement [21]. Upon completion of the pilot study, again through an iterative process, additional feedback was gathered and incorporated into the revised app to further enhance the user experience, before implementing the CRCT. The result was a revised product that incorporated design features, content, and messages, which was relevant for Latina adolescents to increase their understanding of sexual health risk and support them in making informed decisions about contraceptive use.

### Implementing the App Into Clinical Practice

**Figure 1:**
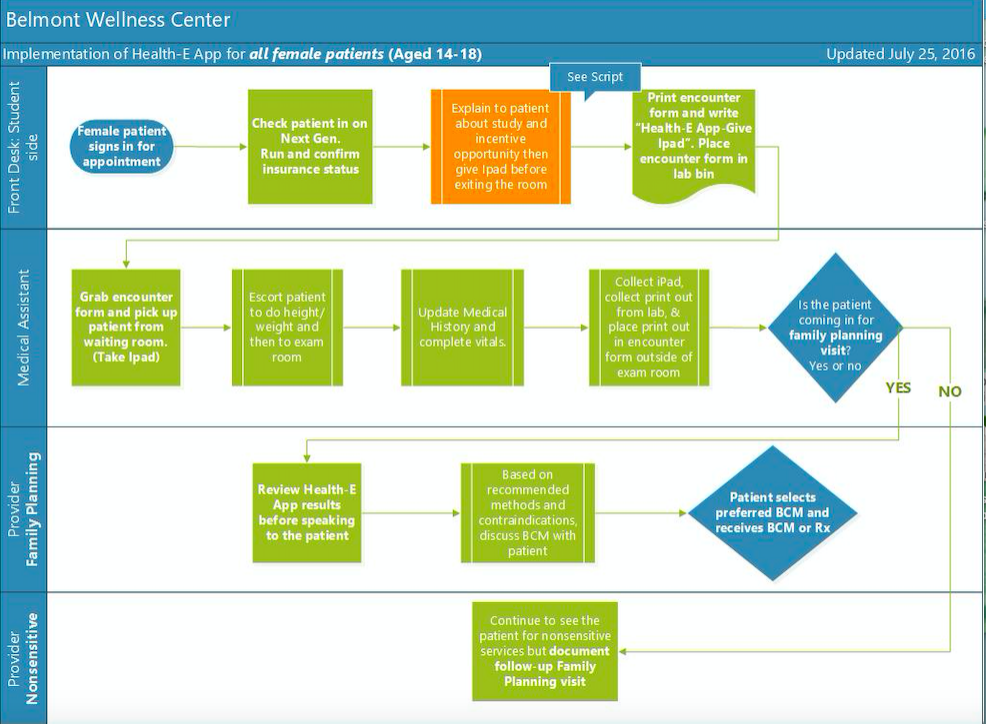
Example of a clinic workflow that integrates the Health-E You app. BCM: birth control method; Rx: prescription.

The implementation of this app into clinical practice was informed by Rogers theory of diffusion of innovation [31,32]. This theory posits that an innovation is more likely to be adopted if it provides value or benefits to potential adopters, fits in well with existing systems, utilizes opinion leaders and champions who can influence others by spreading information about the innovation within and outside the organization, and addresses contextual and managerial factors within an organization [33]. In developing the app, these factors were taken into consideration. Specifically, The LA Trust research associates (RAs) observed the clinic operations and workflows before actual implementation and generated a draft *process map* that detailed how the app would be integrated into each individual clinic’s unique workflow (see Figure 1). The draft process map was shared with each site so that clinic champions and staff could review and discuss the proposed implementation process. After this review, the process map was revised accordingly. This effort helped to ensure that the study team considered the individual needs and perspectives of providers and staff at each clinic with the goal of increasing the likelihood of successful integration and implementation of the app.

In addition, the implementation approaches used in this study were informed by the Patient-Centered Outcomes Research Institute’s (PCORI) 6 principles of engagement: (1) reciprocal relationships between the roles and decision making between the development team, community, and clinical partners; (2) colearning between the research team, community liaisons, and clinical partners; (3) the time and contributions of patients and other stakeholder partners that are valued and demonstrated in fair financial compensation as well as in reasonable and thoughtful requests for their time commitment; (4) transparency; (5) honesty; and (6) trust [34]. As part of the PCORI funding application process, investigators are required to show that their project design adheres to the 6 PCORI principles of engagement. Throughout the life of the project, investigators are asked to self-assess their adherence and provide detailed descriptions of how this study criterion has been met in biannual progress reports. PCORI contract monitors subsequently review the information submitted.

### Data Collection to Assess Implementation of the App

This study used multiple sources of data to assess implementation facilitators and barriers including monthly conference calls, monthly in-person site visits, and quarterly in-person collaborative meetings.

The research team hosted monthly conference calls with The LA Trust and representatives and champions from each of the 9 SBHCs implementing the *Health-E You* app. Meeting notes were summarized and shared with all members of the call and used as a source of data for this study.

In total, 2 community-based, bilingual RAs from The LA Trust, both of whom had prior experience working for or with the various SBHCs, conducted in-person visits at each clinic at least once a month to assess app integration and implementation efforts and to identify technical assistance needs. Immediately after each visit, the RAs completed a data collection form to record implementation approaches, challenges, and technical assistance needs. The RAs provided a summary of each site visit during the research team meetings to identify and resolve any challenges that emerged. A Web-based platform was used to store, organize, and aggregate data for analyses. In addition, The LA Trust team gathered data from the quarterly collaborative quality improvement (QI) meetings to further assess implementation facilitators and barriers. Data were compiled across all 3 sources and analyzed by 2 independent Ras; key themes that emerged across all sites were identified across all sites. Any discrepancies were discussed and resolved by consensus of the research team.

### Overview of App Implementation

Implementation of the app is described in our published study protocol [29]. In brief, clinic staff are to provide all adolescent girls who come into the clinic, for any reason, an iPad, which provides a brief explanation of the research study, obtains consent, and assesses eligibility. Pilot tests indicated that the app took approximately 10-15 min to complete, which could be done while adolescents were waiting to see their clinician.

When app users are done reviewing the various contraceptive methods, they are then asked to select the method(s) they are most interested in using. The app then provides a printout to the clinician before the face-to-face encounter with the patient, and the patient proceeds to the face-to-face encounter with the clinician.

Participants at the control clinics complete a computerized questionnaire to assess their baseline knowledge of contraception and their self-efficacy to obtain sexual health care and use, or nonuse, of contraception before proceeding to their visit to receive care as usual.

## Results

The app was successfully integrated into the clinical workflows of SBHCs, and this study identified a number of factors that supported its integration into clinical workflows, which is described in the following section. In addition, there were a number of implementation challenges, which are also presented.

### Successful Strategies

#### Engagement of Key Stakeholders in App Development

Youth engagement was essential, and youth have been engaged in all aspects of app development and implementation activities. Youth were engaged through the ongoing convenings of The LA Trust Youth Advisory Board (YAB), community advisory boards (CABs), and student advisory boards (SAB) at each clinic site. Specifically, youth guided both the content and design of the app to ensure that it would appeal to their peers. They also provided insights on the reading and comprehension level of the language used in the app. The youth helped develop scripts for video vignettes on each of the contraceptive options and served as actors in these videos. It was then pilot tested at several SBHCs to identify and address barriers to prepare for a larger scale implementation project. Youth, clinic providers, and staff champions were engaged as the project was scaled up and implemented at 9 SBHCs as part of the CRCT. Engaging both youth and clinicians in the app development and iterative pilot testing helped ensure that the ultimate product was developed in a way that was meaningful and of value to both youth and clinicians.

#### Engagement of Key Stakeholders in Implementation Efforts

The LA Trust YAB, CAB, and SABs provided feedback on a regular basis to inform outreach efforts to improve student utilization of the SBHCs and enrollment in the study. Although there were a number of site-specific engagement strategies (posters, student referral cards, lunch timetabling, clinic outreach, etc), the YAB also coordinated women’s health presentations at each of the SBHCs to educate young girls on the services offered at the SBHCs and to stimulate conversations around reproductive health and life planning. On average, approximately 25 students attended each of these presentations. There was usually a clinic staff member present to schedule appointments for interested students.

The engagement of health care providers and clinic staff was equally critical. The RAs identified champions at each site who had a special interest in adolescent health and were excited about using the app. The RAs nurtured these relationships through monthly site visits and regular email and phone communications. They also worked to build relationships with the other providers and staff at each site by expressing a sincere desire to learn what was working well along with the challenges and implementation barriers they faced. The establishment of trust between clinicians, staff, RAs, and researchers enabled SBHC staff and clinicians to feel comfortable in expressing their challenges and concerns. The research team followed up on successes and challenges faced at individual sites through the group QI calls. During these calls, champions from each of the participating SBHCs described their implementation efforts and challenges encountered in a supportive, nonjudgmental context. This process enhanced the disclosure of barriers and allowed for collective problem solving and sharing of strategies that supported more effective implementation.

#### Partnership Structure, Compensation, and Reciprocity

Reciprocity between the university investigators, community partners at The LA Trust, YAB, and clinicians and staff further enhanced implementation efforts. The PCORI contract was awarded to University of California San Francisco (UCSF) but provided fiscal support for the partnerships through a formal subcontract with The LA Trust that supported The LA Trust staff, the YABs, the CABs, and their activities. As a *backbone organization* to support the Wellness Network, The LA Trust facilitates ongoing communications with the field. Learning collaborative meetings were convened quarterly, which brought all stakeholders together so that members could capitalize on each other’s resources and skills and share best practices. These meetings identified topics that were considered important to increasing students’ access to the SBHCs. A number of experts (including the study Principal investigator) were identified and presented on reducing stigma in SBHCs and understanding the state’s minor consent and confidentiality laws. Immediately following each learning collaborative, we held special *strategy meetings* to allow for a more concentrated, site-specific problem-solving session with clinic champions and staff who reviewed study recruitment data and developed QI plans to increase recruitment of Latina patients.

Clinic administrators submitted letters of support articulating the need for and enthusiasm of such a project, strengthening the funding application and reinforcing their commitment to the project. In addition, formal memorandums of understanding (MOUs) were established with each SBHC at the onset of the project to formalize the relationship between the academic partner and community clinic agencies. The MOUs articulated the role of clinic staff members, recruitment goals, and specified the financial stipend each site would receive for their participation. Specifically, mentioning the recruitment goals in the MOU was helpful as a benchmark to discuss individual clinic’s progress toward reaching those goals and as means of discussing implementation barriers and improvement strategies. It also served as a gentle reminder that the success of the project depended on collective efforts and a sense of ownership by all.

It is also important to note that beyond financial compensation, each partner (university and The LA Trust) was valued equally and shared their strengths and expertise with one another through formal presentations, manuscripts, and planning QI efforts to support implementation of the app.

The contributions of the Latina adolescents were also valued and acknowledged through financial stipends, gift cards, and formal acknowledgment of the participation through certificates of participation.

#### Colearning to Support Integration Into Clinical Workflows

To successfully implement a project of this size and scope within the *real-world* clinical setting, understanding the process and flow of the clinic operations was key. Each SBHC had its own unique clinical workflows, procedures, and protocols for serving adolescents, including the provision of reproductive health care. Members of The LA Trust met with the clinic leadership, clinicians, clinic managers, medical assistants, and frontline staff at each SBHC to obtain buy-in, understand the delivery of clinical services for adolescents, and to gather input from all levels of staff feedback on the best ways to integrate the app into each SBHC. As part of the QI process, the team modified the process map to address any unanticipated issues that arose during the initial implementation effort.

Although there was a desire to implement a universal workflow that would ask all female adolescents to participate in the study and for the providers to have a conversation with the student about birth control options, unique workflows had to be established. For instance, some SBHCs were designed to have 2 separate waiting rooms (1 for community patients and 1 for student patients), others had to share 1 congested waiting room for both types of patients. The RAs together with the clinic staff came up with solutions to address the privacy of the students while using the app. For example, 1 SBHC with a shared waiting room had a large health education room that was made available for patients to use the app in privacy, whereas another clinic allowed students to take the iPad with them so they could use the app during vitals.

#### Ongoing Quality Improvement

As noted previously, there were monthly group QI meetings held via WebEx with SBHC champions, The LA Trust team and the UCSF principal investigator and project director. At each QI meeting, the study team provided the SBHCs with data that included the estimated number of youth who visited the clinic (where available), who used the *Health-E You* app, who were eligible to participate in the study, and who ultimately enrolled in the study by site. Using a Plan-Do-Study-Act QI approach [35,36], the study team and SBHCs identified and discussed implementation challenges, successful student engagement and recruitment strategies, and brainstormed ideas for overcoming the challenges. Having staff champions who represent various staff roles (ie, front desk, medical assistants, health educators, and clinic managers) contributed to a holistic discussion of the challenges and successes within and across sites. This approach contributed to increased buy-in and seamless continuity even in the face of staff turnover. Furthermore, this approach also empowered the staff that normally would not be given an opportunity to contribute to research projects of this scale.

#### Transparency, Honesty, and Trust

The UCSF, The LA Trust, and advisory boards worked together through a shared decision-making process. The UCSF and The LA Trust team met on a weekly basis through WebEx meetings to jointly develop the agendas for QI calls, teamwork plans, and action items. SABs met on a quarterly basis and more frequently if needed. There was a strong commitment to open and honest communication with one another, which was exemplified in the QI process.

#### Celebrating Success and Expressing Appreciation

The study team also provided an appreciation party for the youth who helped develop the videos used for the app and distributed participation certificates and *Oscar*-style awards. In addition, many clinicians and staff exceeded our engagement expectations and initiated strategies to improve clinic and app utilization. To express gratitude for this effort, staff was given additional thank-you gifts (eg, gift baskets, lunch for clinic staff, and other tokens of appreciation). These gifts were greatly appreciated and seemed to boost morale and engagement, especially in the context of under-resourced clinical settings where front-line staff and medical assistants may feel under-appreciated for their contributions to clinical operations.

### Challenges

Despite the extensive efforts to support implementation, there were a number of noteworthy challenges.

#### Information Technology Infrastructure: Internet Access and Wireless Connectivity

Technology issues and staff capacity to troubleshoot problems related to technology were the most significant challenges. The amount of technical support and in-person engagement with clinic staff was greater than expected. The vast geographic distance between the SBHCs across Los Angeles made it difficult to identify individual clinic’s ongoing needs or challenges and provide them with immediate support, regular communications, and updates. An online and telephone Technical Assistance support system was implemented to address the logistical challenge of in-person visits. However, this system was not widely used by clinic staff, and issues were more readily identified and addressed via in-person visits.

The app is a Web-based platform, which required Wi-Fi to transfer data captured electronically on the app to the secured back-end data storage system. Before implementing the app, The LA Trust RAs met with the clinic staff to assess technology infrastructure. All sites reported having Wi-Fi; however, the connectivity, reliability, and strength of the signal varied across all sites, and these issues did not come up during the pilot testing phase but emerged as the app was being used across more sites. To address this, the study team purchased mobile Wi-Fi devices (hotspots) to obtain internet access and set up the connection through a local internet service provider. In other cases, we purchased iPads with internet service and set up a data plan with a mobile phone company to access the internet. The specific approach had to be tailored to site-specific needs.

A key feature of the app, to enhance adoption and usability, was the ability for clinicians to see some of the user’s input on the app before the scheduled face-to-face clinic visit. Clinicians prioritized 3 data points for the app to capture and share with them: the contraceptive method(s) the user was interested in, the method(s) the app recommended, and the potential contraindications. Another challenge was developing a system to share data captured from the app with clinicians. It was not possible to directly transmit information from the iPad to the patient’s electronic health record (EHR). At the time of this study, that technology did not exist. Hence, there were multiple EHR systems and some SBHCs used paper charts, thereby, not allowing one uniform system for the transmission of information. In the pilot testing phase, clinicians requested to have this information emailed to them to avoid a paper trail and to move toward an electronic system. However, in developing the clinic workflows for the clinical trial, clinicians at each of these new study sites requested to have the app user’s information printed instead of emails as originally designed, stating that they did not have time or it was not routine to check emails between patients or to use their personal mobile phone. The app had to be redesigned to accommodate this request and it added a new integration challenge. Printers compatible with the iPad were purchased, placed in a private area of the clinic, and set up so they could connect wirelessly to the iPads.

Again, site-specific solutions were necessary to ensure there was a reliable connection to the internet and between the iPad and printers. Even with these solutions in place, several clinics continued to report intermittent problems with connectivity to the internet and the printers’ wireless connections. Few clinics had information technology staff available to address issues that arose; hence, The LA Trust RAs assumed this responsibility.

#### Protecting Patient Confidentiality and Data Security

There was a great deal of concern among providers about protecting patient confidentiality. The data system that stored users’ input was housed on a secure network (encrypted, password-protected, and accessible to only essential research staff). The app informed users that their information was confidential; however, providers and clinic staff in turn needed to remind and assure patients that their information was confidential. In total, 2 sites expressed security concerns about using clinic Wi-Fi for the app and thte protection of patient confidentiality. At 1 site, the MOU needed to be modified to articulate the measures taken to assure data privacy and security (eg, protocol for the protection of patient confidentiality, data encryption, and security of UCSF’s back-end data system), at another we provided our approved protocol of the protection of human use in research.

#### Communication

Despite formal MOUs, monthly site visits, and QI calls, we identified communication barriers between clinic executives and staff that presented additional, unanticipated challenges to app implementation. For example, initially, information about the project was shared with clinic executives (eg, information from MOUs, site incentives, and initial QI data); however, upon conducting site visits, the RAs learned that this information was not universally shared with clinic providers or staff. The LA Trust team had to take extra efforts to ensure all stakeholders received necessary information. In addition, the vast distance between the clinic sites made it difficult to identify and address individual clinic’s technical requirements. The distance did not allow a constant and *real-time* dialogue, even with the use of communication tools such as email and telephone. Often, the staff was too busy or the challenge was not as much a priority as other pressing clinic issues and responsibilities. Hence, these issues were not discovered or addressed until the in-person RA site visits occurred.

#### Staff Turnover

Many SBHCs struggled with high staff turnover, impacting their ability to implement the app. This is a problem not unique to this study and is common with many Federally Qualified Health Centers (FQHCs) who operate clinics in underserved and under-resourced communities [37].

FQHCs are the primary medical sponsors for most of the participating SBHCs in this study. Frequent in-person site visits helped identify staff changes and provide orientation to new staff. In addition, extensive time and effort invested in cultivating relationships with key staff at the clinics improved the extent to which the RAs were informed about staff changes. However, in many situations, staff vacancies remained in place for several months, which impacted the clinic’s capacity to fully utilize the app.

#### Time to Complete App

The app was designed to be completed in approximately 15 min; yet at the same time, it allows for a user-driven educational experience. Actual time to complete the app ranged from 12 to 29 min, with an average completion time of 20 min. As the app is part of a research study, completion time included the time to obtain study consent and participation eligibility. Most clinics adapted their workflows to account for this variation and reported that extra completion time was acceptable because the app helped to *offset* some of the time a clinician would otherwise have to spend providing contraceptive education. Even so, a couple of clinics reported that the time patients needed to complete the app before seeing the clinician remained a challenge, especially during busy clinic hours when patients needed to see the clinician as soon as one became available. The research team with input from the CAB and YAB discussed the possibility of using the app before the clinic visit at home or school, but these approaches were not feasible to implement for a number of reasons. App use outside the clinic setting restricted the ability of providers to receive a printout summary. Providers also did not want to use an alternative form of communication to not disrupt the clinical flow and limit the risk of compromising patient confidentiality.

## Discussion

### Principal Findings

mHealth apps, such as *Health-E You*, have the potential to prepare youth for the clinic visit and act as a clinician extender to support the delivery of health education and enhance the quality of patient-centered sexual health care. Despite the proliferation of mHealth technologies, there is limited research on the integration of mHealth interventions into clinical practice. The purpose of this study was to describe and assess efforts to increase the successful adoption and implementation of the *Health-E You* app at 9 SBHCs that are participating in a larger CRCT.

Implementation of the app was informed by Rogers theory of diffusion of innovation [31,32], PCORI’s principles of engagement, and previous pilot testing, which likely influenced the successful implementation of the app. In particular, engagement of both Latina adolescents and clinic providers helped to create a product (app) that addressed a shared health priority area and the needs of both stakeholders. In addition, other key factors that enhanced integration included significant engagement with clinic staff to meet clinical workflow needs and address technology-related barriers that arose during implementation; engagement with Latina adolescents to identify and address barriers to clinic utilization, promote app usage, and study participation; a strong partnership between university investigators, community partners at The LA Trust, and SBHCs that included formal subcontracts, fiscal support, and a sense of equity, trust, and mutual respect for each other’s roles and expertise; a commitment to understanding local needs and adjusting protocols to fit with clinic specific workflows; an ongoing QI system; regular in-person technical assistance; and celebrating the successes and expressing appreciation for the commitment and effort of the partners, especially clinic staff and youth. We also found that developing strong, positive relationships with front-line clinic staff, medical assistants, and health educators and valuing them as equal partners in the research process was also key to our success.

Although these factors are included in the theory of diffusion of innovation and part of the PCORI principles of engagement, this study was not able to assess specific levels or rates of adoption of the technology. As this is a part of a larger study, there were formal MOUs along with financial incentives to support the participation of SBHCs in the research study, and it is possible that these factors also influenced the willingness of clinics to utilize this technology. As this trial ends and the app becomes available to a wider audience, there will be an opportunity to further assess dissemination, adoption, and diffusion beyond the SBHCs used in this study. There are additional study limitations that should be noted. For instance, this study focused on an app aimed at addressing a specific and sensitive health need for sexually active adolescents who seek care at SBHCs. Thus, findings may not be generalizable to other mHealth apps on different topics or used in other clinic settings. Furthermore, SBHCs are inherently designed to support sexual and reproductive health needs of adolescents and may have been more receptive to the *Health-E You* app than other, less adolescent-friendly, clinical settings.

Despite the strong theoretical foundation and experienced clinical research team, there were a number of implementation challenges. The most significant challenges pertained to limitations with the technology infrastructure of SBHCs, which included intermittent internet access and reliable wireless or mobile phone service connectivity, assuring adolescent confidentiality and data security, communication across multiple levels within a clinic system, staff turnover, and time required for all youth to fully engage in and utilize the multiple features of the app.

### Conclusions

*Health-E You*, an interactive, patient-centered, contraceptive decision support tool, was adopted and integrated into the clinical workflows of all 9 SBHCs. This app was developed to increase adolescents’, who are at a risk of an unintended pregnancy, access to patient-centered contraceptive information and services and was designed to be used in conjunction with a clinical encounter to overcome clinician and individual barriers to comprehensive, patient-centered contraceptive care (including limited time, comfort, and knowledge).

Findings from this study provide important insights that can inform and improve implementation and integration efforts of future mHealth clinical interventions. In particular, this study found that an implementation approach founded in a strong theoretical framework and active engagement with patient and community partners enhanced the development, adoption, and integration of the app into clinical practice. In addition, in implementing mHealth interventions into clinical practice, it is important to consider the perspectives of multiple stakeholders (clinicians, managers, support staff, and patients) and the clinical context to identify strategies that will support the adoption and implementation of technology. It is also important to consider time restrictions, especially in busy clinical practices, and generate alternative ways of leveraging technology within and outside the clinical setting to improve access to health information and services to support health-promoting behaviors among adolescents. In addition, as technology evolves, developing solutions to improve the integration of mHealth technologies with EHR systems is critical.

## Abbreviations

BCM: birth control method
CAB: community advisory board
CRCT: cluster randomized control trial
EHR: electronic health record
FQHCs: Federally Qualified Health Centers
IRB: Institutional Review Board
mHealth: mobile health
MOUs: memorandums of understanding
PCORI: Patient-Centered Outcomes Research Institute
QI: quality improvement
RA: research associate
Rx: prescription
SABs: student advisory boards
SBHC: school-based health center
UCSF: University of California San Francisco
YAB: youth advisory board
